# Effects of an 8-Week Time-Restricted Eating and Walking Exercise on Regional Fat Distribution and Lean Mass in Women with Hidden Obesity: A Randomized Controlled Trial

**DOI:** 10.3390/healthcare14121768

**Published:** 2026-06-18

**Authors:** Shiying Chen, Jakub Kortas, Yulong Ren, Huan Zhou, Haitao Liu

**Affiliations:** 1 Department of Sport, Gdansk University of Physical Education and Sport, 80-336 Gdansk, Poland; shiying.chen@awf.gda.pl (S.C.); jakub.kortas@awf.gda.pl (J.K.); yulong.ren@awf.gda.pl (Y.R.); huan.zhou@awf.gda.pl (H.Z.); 2 College of Physical Education, Henan University, Kaifeng 475001, China

**Keywords:** time-restricted eating, body composition, fat tissue, lean muscle, total body fat, visceral fat

## Abstract

**Objectives**: Explore and compare the effects of 8-week time-restricted eating (TRE), walking exercise, and their combination on fat and lean muscle distribution in female college students with hidden obesity. **Methods**: A total of 68 participants were randomly assigned to four groups: Control (CON), TRE, Exercise (EXE), and TRE + EXE. An 8-week intervention was begun according to a predetermined experimental plan, comparing changes in body fat and lean tissue indices before and after the intervention. **Results**: Before and after the intervention, the TRE group showed a significant decrease in body mass, body mass index (BMI), and total lean mass (*p* < 0.05). The EXE group saw a significant reduction in visceral fat area, visceral fat mass, and visceral fat volume (*p* < 0.01). The TRE + EXE group experienced a significant decrease in android lean mass (*p* < 0.05); Comparing before and after the intervention, there were no statistically significant differences in the body fat percentage, total fat mass, fat and lean in the android and gynoid areas, and %fat in trunk/%fat in legs among the CON, TRE, EXE, and TRE + EXE groups (*p* > 0.05). After the intervention, there were no significant differences in the body fat percentage, total fat mass, total lean mass, fat and lean in the android and gynoid areas, %fat in trunk/%fat in legs, visceral fat area, visceral fat mass, visceral fat volume, subcutaneous fat area, subcutaneous fat mass, and subcutaneous fat volume among the four groups (*p* > 0.05). **Conclusions**: An 8-week TRE intervention in young women with hidden obesity reduced body mass and BMI but also decreased total lean mass, potentially compromising metabolic health, with no statistically significant changes in total body fat or regional fat distribution. Walking exercise showed significant reductions in visceral adiposity indicators (VFA, VFM, VFV), whereas the combined TRE + EXE group did not achieve comparable reductions. These findings suggest that while isolated TRE facilitates body mass loss, it carries a distinct risk of muscle tissue loss and may not confer comparable benefits on visceral fat reduction as walking exercise. However, the generalizability of these preliminary observations is constrained by methodological limitations including retrospective registration, participant attrition, and restricted statistical power. Consequently, these exploratory outcomes must be interpreted with caution, warranting future robust, large-scale trials with enhanced compliance monitoring to optimize prescriptive guidelines for this specific cohort.

## 1. Introduction

The World Obesity Federation considers obesity to be a chronic, relapsing, progressive disease process that requires intervention and control. Consequently, BMI often masks hidden by failing to differentiate between muscle mass and regional body fat accumulation [1]. Fat distribution is a strong and independent predictor of cardiovascular disease, and studies have confirmed that fat quantity in any body region is positively correlated with cardiovascular mortality [2]. The accumulation of visceral fat and central obesity is more closely related to cardiovascular disease risk [3]. Research on body fat distribution can provide a deeper understanding of the causes of obesity and provide valuable information on the development of various comorbidities.

As weight management shifts toward dietary timing, Time-Restricted Eating (TRE) has gained significant attention. According to the latest international consensus, TRE is defined as a dietary regimen in which all caloric intake is confined to a consistent daily window of at least 14 h of fasting, without explicit restriction on energy intake during the eating period [4]. This flexible yet structured dietary pattern, due to its practicality and sustainability, has become increasingly popular in recent years as an effective approach for obesity treatment [5].

Hidden obesity, also alternatively known as normal-weight obesity [6], is a special type of obesity. People with hidden obesity are often defined as having a BMI between 18.5 kg/m^2^ and 23.9 kg/m^2^ and a body fat percentage (BF%) ≥ 30% [7]. Compared to individuals with overweight BMI, people with hidden obesity have a relatively leaner shape [8]. Therefore, people with hidden obesity often do not realize the health risks they face. Crucially, despite their normal weight, NWO individuals exhibit a profoundly elevated cardiometabolic risk profile. Recent evidence indicates they present with significantly higher odds of hyperglycemia and dyslipidemia, rendering their hidden complications highly comparable to overt obesity [9]. Women differ from men in fat distribution, and the incidence of female hidden obesity is 2.5 times that of males [10], hence the choice of the female group as the participants of this study.

TRE confines the entire energy intake to a specific time window within the day [11]. Compared to other fasting methods, TRE requires relatively lower cognitive effort, as individuals do not need to monitor what they eat every day, there is no significant restriction on total energy or diet quality, allowing individuals to maintain their regular diet during the eating window. Thus, the main features of the TRE strategy can be considered simpler or more flexible, hence many people can successfully stick to the TRE diet pattern for longer, thereby promoting long-term weight loss maintenance [12]. Although TRE has been proven to be a safer, more effective, and convenient weight loss dietary strategy [12], most studies only focus on the reduction of overall body weight, not on the specific body parts where weight loss occurs. Due to limited experimental evidence, there is not much research on the effects of TRE intervention on body fat distribution. However, during the weight loss process, it is frequently observed that a diminution in adipose tissue is concomitant with a decrease in lean body mass [13]. In light of this concern, how to preserve lean body mass during dietary interventions has become a growing focus in current research. Within this context, physical activity serves as an important complementary approach to regulating body composition. Several studies have demonstrated that integrating exercise with dietary strategies can promote fat loss while attenuating the reduction in lean tissue. Among various exercise modalities, walking has emerged as a particularly favorable option due to its simplicity, low entry barrier, and superior adherence rates. While dietary restriction alone has been shown to be effective in inducing body mass loss, accumulating evidence suggests that combining it with exercise, particularly sustained low-to-moderate intensity activities such as walking, can lead to superior outcomes in fat reduction and preservation of lean mass [14]. In essence, while TRE facilitates fat loss primarily through energy restriction, the concurrent implementation of walking functions as a vital metabolic countermeasure. Walking exerts sustained mechanical stimuli on the musculoskeletal system, which signals the body to prioritize lean mass retention and effectively counteracts the catabolic state induced by dietary restriction. Consequently, this combined strategy addresses the central paradox of hidden obesity, thereby offering a more effective paradigm for metabolic health optimization than dietary restriction in isolation.

While TRE is widely recognized for its metabolic benefits, controversial findings persist regarding its specific impact on body fat and its distribution [15]. Therefore, this study aims to investigate changes in fat and lean mass content and distribution in female college students with hidden obesity through interventions involving TRE, walking exercise, or their combination, with alterations in total body fat and its distribution designated as the primary endpoints, and changes in lean mass components along with general anthropometric parameters, including body mass and BMI, as the secondary endpoints. We hypothesize that TRE will improve body composition by modulating fat and muscle distribution, and that the TRE + EXE may yield interactive or more favorable effects than either approach alone.

## 2. Methods

### 2.1. Study Design and Participants

**Figure 1:**
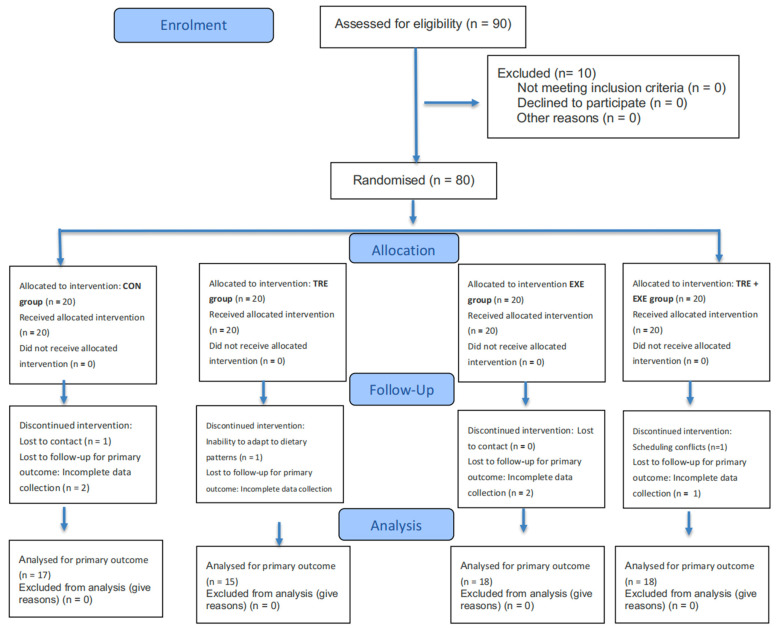
Study recruitment flow chart.

A cohort of 90 female college students (aged 18–22) with hidden obesity was recruited through institutional notifications and campus announcements. Screening for hidden obesity was performed via dual-energy X-ray absorptiometry scanning (Hologic Horizon-Wi, Marlborough, MA, USA). Subjects were enrolled if they met the classification thresholds of a BMI between 18.5 and 23.9 kg/m^2^ and a BF% equal to or greater than 30% (Figure 1) [7]. Exclusion criteria included: (1) pregnancy; (2) participation in structured weight loss programs; (3) use of medications known to affect body composition or metabolism; and (4) any condition that could interfere with the accurate assessment of body composition or fat distribution. Ultimately, 80 eligible participants were selected. The participants were randomly divided into four groups using a random number table method. To ensure allocation concealment, sequentially numbered, opaque sealed envelopes were used. After 8 weeks of intervention, one person dropped out due to personal reasons, one person dropped out due to not completing the data collection after the intervention, one person dropped out due to inability to adapt to the TRE strategy, and nine people were excluded due to incomplete test data items. Ultimately, data from 68 participants were included in the analysis, including CON group (N = 17), TRE group (N = 15), EXE group (N = 18), TRE + EXE group (N = 18) (Figure 1).

Before entering the study, the participants completed a screening examination, including body composition testing and a questionnaire survey. Body composition testing assessed values such as body fat and muscle mass to assess the participants’ physical condition and ensure they met the study’s physiological criteria. The questionnaire collected information on the participants’ health history, lifestyle habits, and previous illnesses, helping to identify individuals with conditions that could impact the study results. By implementing these measures, researchers can exclude individuals who may affect study outcomes, thus ensuring the validity and reliability of research data. This study has been approved by Ethics Committee of Human Research of Henan university (HUSOM2021-210 on 6 September 2021). The trial protocol was publicly registered at the Chinese Clinical Trial Registry (registration number: ChiCTR2600125017 on 20 May 2026). The trial was registered retrospectively; this registration delay was due to the authors’ initial unfamiliarity with the concurrent international clinical trial registry workflow. The research was performed in accordance with relevant guidelines. All participants were informed of the purpose of the study and the possible risks of the experiment before participating in the project. Each participant signed an informed consent form. At the end of the experiment, they were each provided with a personalized health report.

### 2.2. Experimental Intervention

The intervention period was 8 weeks. During the intervention period, all participants maintained a free-living status and selected their own food. Based on relevant experimental designs [16], participants in the CON group were instructed to preserve their habitual lifestyle. The TRE group adopted 8 h TRE (libitum eating from 10:00–18:00 daily, fasting at other times) with no step count target. The EXE group was required to accumulate a daily step count of 11,000–12,499 steps. Participants in the TRE + EXE group needed to meet both the 8 h TRE and 11,000–12,499 steps. A triaxial accelerometer was employed to quantify participants’ daily walking activity.

### 2.3. Measurements

#### 2.3.1. Height

For stature assessment, subjects stood erect and barefoot on a stadiometer with their heels touching and forefeet abducted at a 60-degree angle. Height was recorded to the nearest 0.01 cm. All measurements were performed in triplicate, and the mean value was utilized for analysis.

#### 2.3.2. Body Composition

Whole-body Dual-energy X-ray Absorptiometry (DXA) can accurately and precisely assess body fat mass in different parts of the body, which has been fully verified [17]. Therefore, this study used DXA to assess participants’ body mass, BMI, BF%, total fat mass (TFM, kg), total lean mass (TLM, kg), android %fat/gynoid %fat (A/G), percent fat in android area (Android %fat), percent fat in gynoid area (Gynoid %fat), android fat mass (AFM, kg), gynoid fat mass (GFM), android lean mass (A-LM, kg), gynoid lean mass (G-LM, kg), %fat in trunk/%fat in legs (T/L %fat), Visceral fat area (VFA, cm^2^), visceral fat mass (VFM, kg), visceral fat volume (VFV, L), subcutaneous fat area (SFA, cm^2^), subcutaneous fat mass (SFM, kg), subcutaneous fat volume (SFV, L) for testing. As the primary interest was in assessing adipose and lean tissue changes across different body regions, bone mineral content was not included in the body mass calculations. Total body mass was computed as the aggregate of TFM and TLM. Participants were aligned centrally in a supine position on the examination bed, ensuring the head was placed approximately 3 cm inferior to the scan line. Arms were extended alongside the body with palms facing downward and fingers slightly spread. Feet were positioned in an inward “V” shape, with big toes together and heels apart. The entire body was kept within the scanning field. During the scan, participants remained still while the scanner moved from the head to the lower limbs. Data were automatically analyzed using the system’s software. All participants were instructed to wear short sleeves and shorts and to remove any metal accessories such as rings or necklaces before the scan. They were also required to undergo the assessment during the morning after an overnight fasting period lasting 8 h or more and to void their bladder immediately prior to scanning, ensuring standardized physiological conditions. During the procedure, participants lay in the anatomical position and were asked to remain as still as possible to avoid motion artifacts that might impair data accuracy. All scans were performed using the same Hologic Horizon Wi system, operated by a single trained technician to ensure procedural consistency.

The device was calibrated daily using a standard spine phantom to ensure accuracy. Scans were analyzed with APEX (v5.6.0). ROI lines for android, gynoid, visceral, and subcutaneous regions were auto-generated and manually adjusted as needed.

#### 2.3.3. Step

The ActiGraph GT9X Link is widely used and often regarded as a criterion device for validating physical activity trackers, including step counting and energy expenditure metrics in research studies. Recent systematic reviews have described it as a “gold-standard” activity monitor for such purposes [18]. In this study, we used the ActiGraph GT9X Link to track each participant’s daily step counts.

Researchers directed participants to carry it continuously on the their left wrist, removing it only for bathing and swimming. The average daily step counts for each group throughout the 8-week intervention (Table 1). Based on Table 1 and the step count classification proposed by Tudor-Locke et al., participants in the CON and TRE groups (5000–7499 steps/day) were classified as low active, while those in the EXE and TRE + EXE groups (≥10,000 steps/day) met the criteria for active. This classification reflects clear differences in baseline physical activity levels across groups, which may influence intervention outcomes [19].

#### 2.3.4. Experimental Control

Before the experiment started, centralized lectures were given to the participants of each group on the detailed implementation of the TRE method and walking exercise plan to ensure that each participants could implement it themselves. Group leaders were established for each group to manage the participants’ eating times and exercise conditions. The CON group was required to maintain their usual physical activity levels and dietary patterns throughout the experiment period.

#### 2.3.5. Sample Size

G*power software (version: 3.1.9.2) was employed for sample size estimation. Under the conditions of α = 0.05, effect size = 0.45, and four groups (one-way ANOVA, fixed effects, omnibus), at least 65 participants were needed, with a statistical test power reaching 85%. The ultimate cohort size utilized for final analysis in this study was sixty-eight, meeting the research needs. The chosen effect size (f = 0.45) corresponds to a moderate-to-large effect and was informed by findings from previous studies investigating the effects of time-restricted eating, with or without exercise, on body composition parameters [20].

#### 2.3.6. Statistical Analysis

SPSS 25.0 software was utilized to perform statistical evaluations on the experimental dataset, with descriptive statistics expressed as mean ± standard deviation (X ± SD). Baseline comparability across the four groups was examined using a one-way analysis of variance (ANOVA); Longitudinal intra-group variations were evaluated via paired t-tests; and between-group differences at post-intervention were assessed with one-way ANOVA, with least significant difference (LSD) post hoc comparisons applied when the omnibus F test was significant. All tests were two-sided. Statistical significance was designated at *p* < 0.05, while a threshold of *p* < 0.01 defined high significance.

#### 2.3.7. Use of AI-Assisted Tools

The authors used AI-assisted language tools ChatGPT, version ChatGPT-4o, OpenAI, San Francisco, CA, USA; Grammarly, desktop version, Grammarly Inc., San Francisco, CA, USA to edit wording and improve readability. These tools were not involved in the generation of data, results, or interpretations. All sections were critically reviewed and approved by the authors.

## 3. Results

### 3.1. Fat and Lean Tissue

As shown in Table 2, following the 8-week trial, the TRE group participants’ body mass, BMI, and TLM significantly decreased (*p* < 0.05); the CON group’s BMI significantly decreased (*p* < 0.05). In contrast, TBF, TFM, TLM, T/L %fat remained unchanged within the CON, EXE, and TRE + EXE groups (*p* > 0.05).

### 3.2. Android and Gynoid Area

After the 8-week of intervention, the TRE + EXE group participants’ A-LM significantly decreased (*p* < 0.05), while the CON, TRE, and EXE group A-LM did not change over the intervention (*p* > 0.05). The A/G, Android %fat, AFM, Gynoid %fat, GFM, G-LM remained unaltered throughout the trial across the CON, TRE, EXE, and TRE + EXE groups (*p* > 0.05) (Table 3).

### 3.3. Visceral and Subcutaneous Area

**Figure 2:**
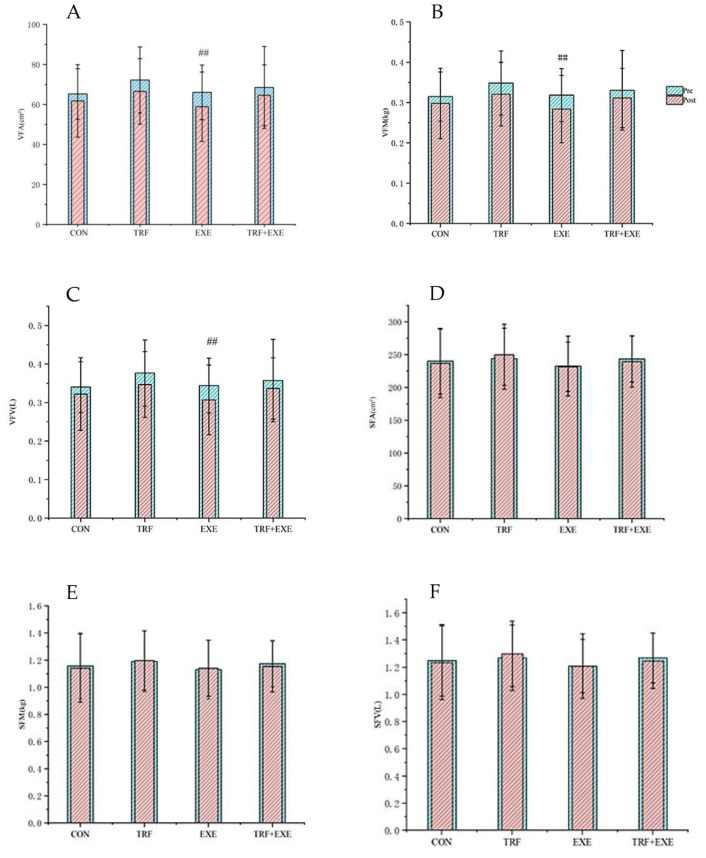
Pre-intervention visceral fat indices and post-intervention visceral fat indices at 8 weeks. Data are presented as mean ± SD. (**A**) VFA, visceral fat area; (**B**) VFM, visceral fat mass; (**C**) VFV, visceral fat volume; (**D**) SFA, subcutaneous fat area; (**E**) SFM, subcutaneous fat mass; (**F**) SFV, subcutaneous fat volume. ## indicates *p* < 0.01. CON, control; TRE, time-restricted eating; EXE, exercise; TRE + EXE, time-restricted eating combined with exercise.

After 8 weeks of intervention, within-group comparisons found that the EXE group’s VFA, VFM, VFV significantly decreased (*p* < 0.01), while the CON, TRE, and TRE + EXE groups did not change over the intervention (*p* > 0.05). The SFA, SFM, SFV values remained unchanged across the CON, TRE, EXE, and TRE + EXE groups (*p* > 0.05) (Figure 2).

### 3.4. Comparison of Groups Post-Intervention

After the 8-week intervention, between-group comparisons showed no statistically significant difference in post-intervention test values among the CON group, TRE group, EXE group, and TRE + EXE groups (*p* > 0.05) (Table 4).

## 4. Discussion

The present investigation evaluated how an 8-week TRE protocol influences adiposity and muscular tissue distribution among young women with hidden obesity, utilizing DXA for pre- and post-intervention assessments. Additionally, the implementation of a randomized controlled framework yielded a robust level of scientific evidence, including a control group that did not change their daily diet and exercise habits, as well as intervention groups that employed only TRE, only exercise, and a combination of TRE and exercise. The results show that exercise can reduce visceral fat values in female college students with hidden obesity. TRE can reduce weight, BMI, and TLM in these students, but it does not significantly affect the distribution of body fat.

Dietary intervention is a primary treatment method for obesity, with each dietary adjustment primarily focused on calorie restriction and consequent weight loss. This study shows that TRE can effectively control and reduce the weight and BMI of female college students with hidden obesity, consistent with prior research results both domestically and internationally [21]. Initial weight loss stems from a caloric deficit, as this regimen prompts subjects to spontaneously reduce energy intake.

Body composition distribution reflects individual health. Muscle and fat, as important body components, participate in body metabolism, and changes in the quality of these tissues can indirectly reflect the metabolic state of the body. The effects of TRE on adiposity and muscular mass remain highly debatable [22]. This study demonstrates that TRE significantly reduced muscle mass in participants. Though statistically notable, this minor fat loss (~0.7 kg) carries limited clinical significance for an 8-week trial. This finding contrasts with some studies conducted in non-obese populations, where TRE did not lead to significant losses in lean body mass, particularly when physical activity was maintained or protein intake was sufficient [23]. However, other studies, including those involving normal-weight or overweight individuals, have reported that intermittent fasting regimens may reduce TLM, especially in the absence of resistance training or dietary control [24]. Variations in subject profiles, trial lengths, and exercise designs likely explain these conflicting outcomes. Although some studies have shown that TRE can prevent the decline in muscle function caused by obesity [25], other studies indicate that intermittent fasting reduces TLM [26], which our study also confirms. The sex and age of the subjects, as well as the precise composition of the food, were equally important for the study. Animal models show that TRE outcomes heavily depend on sex [27], making gender a crucial determinant of lean mass [28]. Specifically, TRE protects muscular function and coordination solely in male mice [29]. Female college students with hidden obesity exhibit unhealthy trends in dietary habits and physical exercise routines. TRE shifts feeding windows without altering dietary composition or physical habits, inherently creating an energy deficit [22], with lack of exercise and poor nutrition being common factors in the reduction in muscle mass. Consequently, combining TRE with resistance training or tailored physical activity is a crucial strategy for young women to enhance physical performance while preserving functional tissue structure [22,30]. Therefore, in future interventions, the combination of TRE with exercise could be considered to enhance the benefits of TRE.

Elevated torso fat and reduced leg fat both heighten cardiovascular risks. An increase in trunk fat is a direct predictor of increased incidence of cardiovascular disease, while an increase in leg fat can prevent glucose metabolic disorders, especially in women [30]. These insights underscore how regional fat distribution dictates cardiometabolic health.

BF% reflects general obesity, while the A/G reflects the distribution of body fat. Increasing evidence suggests that regional fat accumulation reflects health risks better than total fat volume [31]. A single DXA scan provides a reliable and precise assessment of body composition, including the regional distribution of fat and lean tissue. Android fat, which is primarily distributed across the upper body and torso, including the abdomen and chest, forms an “apple-shaped” body or central obesity, and android adiposity mirrors cardiovascular risks more sensitively [32]. Gynoid fat, which forms around the buttocks, breasts, and upper thighs, is more likely stored in the lower body in women [31]. Hence gynoid fat is also called “female fat,” forming a “pear-shaped” body, with relatively lower risk. Gynoid fat has a protective role, while android fat has harmful effects [33]. While excess fat triggers metabolic issues, preserving gynoid fat might protect cognitive function [34]. Our study results show that 8 weeks of TRE and walking intervention did not alter android or gynoid fat in participants. However, combining these two strategies significantly reduced android area lean tissue. Although studies show that calorie restriction can reduce visceral and subcutaneous tissues [35], other studies indicate that changes in lifestyle and diet are not sufficient to achieve significant weight loss, especially in the abdominal area [36], However, adjustments in dietary structure have been shown to also reduce android area lean tissue. It is worth mentioning that lean tissues in different areas have varying impacts on overall cardiovascular mortality. A-LM and G-LM are better indicators for predicting mortality and cardiovascular-related mortality, with A-LM positively correlated with cardiovascular mortality. According to reports, Exercise effectively combats obesity and affects muscle gain, with increased physical activity closely related to abdominal fat distribution [37]. Studies have shown that at least 8 weeks of moderate to high-intensity exercise is effective in reducing abdominal fat [38], and increasing muscle mass and physical activity can also offset the negative effects of visceral fat.

Abundant evidence links visceral adiposity more closely to cardiovascular risks than SAT. with weight loss, visceral fat tissue is preferentially reduced compared to SAT. SAT is commonly assessed using three indicators: SFA, SFM, and SFV, which together reflect the distribution and amount of subcutaneous fat deposits. However, some studies have shown that changes in diet quality are only statistically significant in association with visceral fat tissue in men [39]. Notably, the observed reduction in visceral adiposity indicators (VFA, VFM, VFV) in the EXE group aligns with previous evidence demonstrating that moderate aerobic exercise effectively decreases visceral fat stores [40]. Beyond mere energy expenditure, walking serves as a dual-action metabolic modulator. It provides consistent mechanical stimuli to the musculoskeletal system, which is essential for lean mass preservation, while concurrently accelerating systemic lipid oxidation to facilitate VAT mobilization. This mechanism effectively addresses the central paradox of hidden obesity: the requirement to mitigate visceral adiposity while simultaneously safeguarding functional muscle mass [41]. In contrast, the TRE + EXE group did not exhibit comparable improvements. Concurrently, while TRE drives weight loss, it simultaneously reduces TLM [42], which may attenuate the metabolic benefits typically conferred by exercise. Previous studies, including our own prior research [43], have demonstrated that TRE may lead to losses in TLM and elevations in blood lipid levels in young women with hidden obesity, reflecting a potential catabolic response. Because TLM represents skeletal muscle mass and drives insulin-mediated glucose uptake and lipid oxidation, reductions in TLM may impair metabolic flexibility and diminish VAT mobilization in response to physical activity. The modest effect sizes observed further highlight that meaningful improvements in body composition may require either longer intervention periods or higher training load.

SAT is the main part of body fat and has lower activity compared to visceral fat. Both visceral and subcutaneous fat tissues are related to cardiovascular risk, unrelated to overall obesity. Men and women have different body fat distributions, with men’s fat mostly distributed in the viscera and adult women preferentially storing energy in subcutaneous fat tissue, thus women’s fat is mostly distributed subcutaneously. While weight loss can have a positive impact on subcutaneous fat tissue, high accumulation of subcutaneous fat can hinder weight loss in women. In our study, TRE did not produce significant changes in SAT among young women with hidden obesity. This finding aligns with recent randomized controlled trials reporting that short- to medium-term TRE interventions failed to significantly reduce SAT, even when body mass declined or metabolic parameters improved [44]. These results suggest that TRE alone may exert limited effects on subcutaneous fat depots, particularly in the absence of structured dietary control or targeted physical activity.

Taken together, while the intervention induced statistically significant changes, the relatively small magnitude limits immediate clinical translation. Nevertheless, this study provides valuable preliminary evidence and pilot data to inform the design of longer-term trials integrating both TRE and exercise interventions in female with hidden obesity.

## 5. Limitation

This study has limitations. It was an 8-week, single-center trial in young women with normal-BMI obesity, which limits generalizability. Only baseline and post-intervention assessments were collected, reducing sensitivity to small short-term changes. Free-living diet and physical activity outside the protocol were not systematically monitored, and adherence to the eating window/exercise dose was not objectively captured, which may have attenuated between-group effects. Between-group inference relied on post-intervention one-way ANOVA together with within-group paired t-tests; although baseline comparability was verified, baseline-adjusted or mixed-effects models might be more efficient. Finally, DXA provides estimated body composition values with a typical error of ~2–3%, which should be considered when interpreting small effects. Additionally, the lack of physical fitness measures, such as cardiorespiratory fitness or muscular strength, further limits the generalizability of our findings to broader functional adaptations. Future studies should include longer follow-up, intermediate assessments, objective adherence and diet monitoring, and model-based analyses.

## 6. Conclusions

This study found that 8 weeks of time-restricted eating can reduce the body mass, BMI, and total lean mass, but TRE does not significantly affect the body fat distribution of female college students with hidden obesity. The EXE group showed significant reductions in visceral adiposity indicators (VFA, VFM, VFV), whereas the combined TRE + EXE group did not achieve comparable reductions. These findings suggest that while TRE facilitates body mass loss, it may not confer comparable benefits on lean mass preservation or fat redistribution as walking exercise.

## Figures and Tables

**Table 1:** Average daily walking numbers during the 8-week intervention.

Groups	Daily Walks (Steps)
CON	7065 ± 2205
TRE	6544 ± 1984
EXE	11,587 ± 1355
TRE + EXE	12,328 ± 1537

Data are presented as mean ± SD.

**Table 2:** Comparison of overall whole-body test indicators, trunk and leg fat indicators before and after intervention.

Index	CON (N = 17)			TRE (N = 15)			EXE (N = 18)			TRE + EXE (N = 18)		
	Pre	Post	*p*	Pre	Post	*p*	Pre	Post	*p*	Pre	Post	*p*
Weight (kg)	54.64 ± 5.14	54.71 ± 5.07	0.86	55.98 ± 4.63	55.06 ± 4.52	0.03 *	53.39 ± 5.26	53.14 ± 5.47	0.41	55.46 ± 4.51	54.80 ± 5.11	0.20
BMI (kg/m^2^)	20.36 ± 1.52	20.38 ± 1.48	0.87	21.21 ± 1.34	20.86 ± 1.25	0.03 *	20.61 ± 1.46	20.51 ± 1.47	0.36	21.35 ± 1.47	21.11 ± 1.93	0.23
TBF (%)	38.38 ± 3.69	38.17 ± 4.65	0.61	38.13 ± 3.42	38.66 ± 3.18	0.25	38.26 ± 2.68	38.75 ± 2.78	0.13	38.93 ± 2.32	39.05 ± 2.67	0.73
TFM (kg)	21.11 ± 3.74	21.04 ± 4.12	0.78	21.41 ± 3.12	21.32 ± 2.67	0.70	20.46 ± 2.80	20.63 ± 2.96	0.44	21.63 ± 2.54	21.47 ± 3.00	0.49
TLM (kg)	33.53 ± 2.12	33.67 ± 2.33	0.70	34.57 ± 2.61	33.74 ± 2.95	0.01 **	32.94 ± 3.16	32.51 ± 3.24	0.07	33.83 ± 2.53	33.33 ± 2.67	0.22
T/L %fat	0.85 ± 0.06	0.84 ± 0.05	0.48	0.89 ± 0.09	0.88 ± 0.08	0.36	0.83 ± 0.09	0.83 ± 0.09	0.91	0.86 ± 0.08	0.85 ± 0.08	0.79

Data are presented as mean ± SD. Pre, pre-intervention; Post, post-intervention; Weight represents the sum of TFM and TLM (soft tissue mass) as assessed by DEXA. Bone mineral content (BMC) was not included; BMI, body mass index; TBF, total body fat; TFM, total fat mass; TLM, total lean mass; T/L %fat, %fat in trunk/%fat in legs. * indicates *p* < 0.05, ** indicates *p* < 0.01.

**Table 3:** Comparison of test indicators in the Android and Gynoid areas before and after intervention.

Index	CON (N = 17)			TRE (N = 15)			EXE (N = 18)			TRE + EXE (N = 18)		
	Pre	Post	*p*	Pre	Post	*p*	Pre	Post	*p*	Pre	Post	*p*
A/G	0.87 ± 0.08	0.88 ± 0.08	0.57	0.89 ± 0.10	0.90 ± 0.10	0.33	0.87 ± 0.09	0.87 ± 0.08	0.84	0.86 ± 0.07	0.87 ± 0.08	0.31
Android %fat	37.52 ± 4.86	37.24 ± 5.78	0.67	37.87 ± 4.63	38.55 ± 5.01	0.28	37.33 ± 4.28	37.47 ± 3.96	0.75	37.30 ± 3.39	37.70 ± 4.07	0.44
AFM (kg)	1.37 ± 0.33	1.36 ± 0.39	0.82	1.38 ± 0.28	1.40 ± 0.32	0.68	1.27 ± 0.26	1.26 ± 0.28	0.54	1.37 ± 0.26	1.32 ± 0.29	0.11
ALM (kg)	2.24 ± 0.22	2.24 ± 0.26	0.99	2.23 ± 0.17	2.19 ± 0.22	0.26	2.11 ± 0.16	2.08 ± 0.27	0.43	2.27 ± 0.17	2.16 ± 0.26	0.02 *
Gynoid %fat	42.89 ± 3.31	42.19 ± 4.37	0.21	42.57 ± 3.00	42.60 ± 2.94	0.95	42.69 ± 2.90	42.93 ± 2.49	0.53	43.48 ± 2.11	43.43 ± 2.89	0.91
GFM (kg)	3.96 ± 0.66	3.93 ± 0.75	0.62	3.90 ± 0.41	3.92 ± 0.40	0.74	3.87 ± 0.53	3.88 ± 0.52	0.87	4.12 ± 0.47	4.05 ± 0.53	0.17
GLM (kg)	5.24 ± 0.44	5.33 ± 0.41	0.26	5.26 ± 0.43	5.28 ± 0.46	0.75	5.18 ± 0.54	5.15 ± 0.61	0.64	5.33 ± 0.45	5.27 ± 0.63	0.52

Data are presented as mean ± SD. Pre, pre-intervention; Post, post-intervention; A/G, android %fat/gynoid %fat; Android %fat, percent fat in android area; AFM, android fat mass; ALM, android lean mass; Gynoid %fat, percent fat in gynoid area; GFM, gynoid fat mass; GLM, gynoid lean mass. * indicates *p* < 0.05.

**Table 4:** Post-intervention comparison of body composition values among groups.

Index	CON (N = 17)	TRE (N = 15)	EXE (N = 18)	TRE + EXE (N = 18)	*p*
Body mass (kg)	54.71 ± 5.07	55.06 ± 4.52	53.14 ± 5.47	54.80 ± 5.11	0.68
BMI (kg/m^2^)	20.38 ± 1.48	20.86 ± 1.25	20.51 ± 1.47	21.11 ± 1.93	0.51
TBF (%)	38.17 ± 4.65	38.66 ± 3.18	38.75 ± 2.78	39.05 ± 2.67	0.90
TFM (kg)	21.04 ± 4.12	21.32 ± 2.67	20.63 ± 2.96	21.47 ± 3.00	0.88
TLM (kg)	33.67 ± 2.33	33.74 ± 2.95	32.51 ± 3.24	33.33 ± 2.67	0.56
T/L %fat	0.88 ± 0.09	0.89 ± 0.11	0.85 ± 0.10	0.85 ± 0.11	0.53
A/G (%)	0.88 ± 0.08	0.90 ± 0.10	0.87 ± 0.08	0.87 ± 0.08	0.63
Android %fat	37.24 ± 5.78	38.55 ± 5.01	37.47 ± 3.96	37.70 ± 4.07	0.88
AFM (kg)	1.36 ± 0.39	1.40 ± 0.32	1.26 ± 0.28	1.32 ± 0.29	0.65
A-LM (kg)	2.24 ± 0.26	2.19 ± 0.22	2.08 ± 0.27	2.16 ± 0.26	0.31
Gynoid %fat	42.19 ± 4.37	42.60 ± 2.94	42.93 ± 2.49	43.43 ± 2.89	0.72
GFM (kg)	3.93 ± 0.75	3.92 ± 0.40	3.88 ± 0.52	4.05 ± 0.53	0.83
G-LM (kg)	5.33 ± 0.41	5.28 ± 0.46	5.15 ± 0.61	5.27 ± 0.63	0.79
VFA (cm^2^)	61.77 ± 18.09	66.13 ± 16.88	57.70 ± 17.04	64.20 ± 15.56	0.51
VFM (kg)	0.30 ± 0.09	0.32 ± 0.08	0.28 ± 0.08	0.31 ± 0.07	0.51
VFV (L)	0.32 ± 0.09	0.34 ± 0.09	0.30 ± 0.09	0.33 ± 0.08	0.51
SFA (cm^2^)	236.75 ± 52.07	249.82 ± 46.48	231.56 ± 37.65	236.85 ± 38.49	0.68
SFM (kg)	1.14 ± 0.25	1.20 ± 0.22	1.12 ± 0.18	1.14 ± 0.19	0.68
SFV (L)	1.23 ± 0.27	1.30 ± 0.24	1.21 ± 0.20	1.23 ± 0.20	0.68

Data are presented as mean ± SD. BMI, body mass index; TBF, total body fat; TFM, total fat mass; TLM, total lean mass; T/L %fat, %fat in trunk/%fat in legs; A/G, android %fat/gynoid %fat; Android %fat, percent fat in android area; AFM, android fat mass; A-LM, android lean mass; Gynoid %fat, percent fat in gynoid area; GFM, gynoid fat mass; G-LM, gynoid lean mass; VFA, visceral fat area; VFM, visceral fat mass; VFV, visceral fat volume; SFA, subcutaneous fat area; SFM, subcutaneous fat mass; SFV, subcutaneous fat volume.

## Data Availability

The data presented in this study are available on request from the corresponding author. The data are not publicly available due to privacy and ethical restrictions.
